# Effect of augmented reality navigation technology on perioperative safety in partial nephrectomies: A meta-analysis and systematic review

**DOI:** 10.3389/fsurg.2023.1067275

**Published:** 2023-04-12

**Authors:** Cong Cheng, MaCheng Lu, Ye Zhang, XingQian Hu

**Affiliations:** Department of General Surgery, Wuxi People’s Hospital Affiliated to Nanjing Medical University, Wuxi, China

**Keywords:** partial nephrectomy, radical nephrectomy, nephron-sparing surgery, perioperative safety, augmented reality navigation technology

## Abstract

**Aim:**

To evaluate the impact of augmented reality surgical navigation (ARSN) technology on short-term outcomes of partial nephrectomy (PN).

**Methods:**

A systematic literature search was conducted in PubMed, Embase, Cochrane, and Web of Science for eligible studies published through March 28, 2022. Two researchers independently performed the article screening, data extraction and quality review. Data analysis was performed using Cochrane Review Manager software.

**Results:**

A total of 583 patients from eight studies were included in the analysis, with 313 in the ARSN-assisted PN group (AR group) and 270 in the conventional PN group (NAR group). ARSN-assisted PN showed better outcomes than conventional surgery in terms of operative time, estimated blood loss, global ischemia rate, warm ischemia time, and enucleation rate. However, there were no significant differences in the rate of Conversion to radical nephrectomy (RN), postoperative estimated glomerular filtration rate (eGFR), positive margin rate, and postoperative complication rate.

**Conclusion:**

The utilization of ARSN can improve the perioperative safety of PN. Compared with conventional PN, ARSN-assisted PN can reduce intraoperative blood loss, shorten operative time, and improve renal ischemia. Although direct evidence is lacking, our results still suggest a potential advantage of ARSN in improving renal recovery after PN. However, as the ARSN system is still in an exploratory stage, its relevance in PN have been poorly reported. Additional high-quality randomized controlled trial (RCT) studies will be required to confirm the effect of ARSN on PN.

**Systematic Review Registration:**

https://www.crd.york.ac.uk/prospero/display_record.php?RecordID=301798, identifier PROSPERO ID: CRD42022301798.

## 1. Introduction

Over the past decade, nephron-sparing surgery (NSS) has gradually become the preferred surgical approach for cT1a tumors (1). Compared to radical nephrectomy (RN), partial nephrectomy (PN) preserves more of the renal parenchyma and improves recovery of renal function after surgery (2).

In NSS for endophytic renal masses, the tumor cannot be localized by observing the kidney surface, thus the treatment outcome largely depends on the surgeon's competence and experience (3). In addition, surrounding normal kidney tissue is often sacrificed to ensure border security. Furthermore, when adherent perirenal fat (APF) is present, the surgical field is disturbed and the operative area is compressed, making the surgery more difficult (4). Over the past two decades, different surgical navigation techniques have been adopted for NSS to improve surgical safety. Currently, the most commonly employed navigation modalities are intraoperative ultrasound (IOUS) and fluorescence guidance (5–8).

Consistent with the advancement of image guidance technology in recent years, augmented reality (AR) technology has gradually been applied in NSS's navigation (9–11). Its approach is based on reconstructing a three-dimensional kidney model (3D kidney model) based on the imaging data (CT/MRI, etc.) of the patient's kidney before surgery and then superimposing the model on the actual surgical area for intraoperative navigation (11). The preoperative 3D kidney model can directly display the course of vessels and ureters, as well as the location and shape of the tumor. By carefully investigating the kidney model and performing virtual surgery, the surgeon can obtain detailed anatomical information about the surgical area, which helps to refine the preoperative strategy (12, 13). Intraoperatively, the surgeon can directly view the position of the tumor and its surrounding anatomical features using a registered kidney model. Many studies on ARSN have indicated its great potential for use in surgical procedures (6, 14–19). Concerning PN, several previous studies have reported the advantages of ARSN-assisted PN over the conventional approach (6, 20–26). However, the findings were not entirely consistent across studies. Therefore, we conducted this meta-analysis to systematically evaluate the effect of ARSN on the efficacy and safety of PN surgery.

## 2. Materials and methods

### 2.1. Search strategies

Two researchers independently searched PubMed, Embase, Cochrane Library, and Web of Science. All studies up to March 28, 2022 were searched according to the following search strategy: (((Nephrectomy [MeSH Terms]) OR (Heminephrectom*)) OR (Nephrectom*)) AND (((((((((Augmented reality [MeSH Terms]) OR (Augmented Realit*)) OR (Realit*, Augmented)) OR (Mixed Reality)) OR (Mixed Realit*)) OR (Realit*, Mixed)) OR (Reality, Mixed)) OR (image-guided surgery)) OR (IGS)).

PROSPERO (International Prospective Register of Systematic Reviews) number CRD42022301798 was used to register this study.

### 2.2. Inclusion and exclusion criteria

The inclusion criteria are as follows: (1) Population: Patients requiring PN; (2) Intervention: PN guided by ARSN system; (3) Comparison: PN guided by preoperative imaging (CT/MRI), IOUS, or fluorescence imaging; (4) Outcomes: estimated blood loss (EBL), operative time (OT), postoperative complication, warm ischemic time (WIT), eGFR, and rate of enucleation, global ischemic, positive surgical margin and conversion to RN.

Exclusion criteria: (1) Duplicate publications by the same author or institution. (2) Non-comparative research, such as case reports, and cross-sectional studies.

Two researchers independently scanned the titles and abstracts for initial screening. Then, carefully read the full text in order to define whether each of them was eligible for the analysis. Upon differences that could not be resolved after discussion, a third author was consulted.

### 2.3. Data extraction

Two researchers collected pertinent data separately. The data would be reviewed to ensure correctness. Data extracted from each paper included: the author's name(s), year and country of publication, number of participants, patient's age, gender, body mass index (BMI), tumor size, Padua and R.E.N.A.L score, and outcome data.

Major outcomes include operative time (OT), estimated blood loss (EBL), warm ischemic time (WIT), and eGFR changes (Postoperative, 3-month postoperative, and 6-month postoperative data); Minor results include postoperative complication (Clavian-Dindo classification), and rate of enucleation, global ischemic, positive surgical margin and conversion to RN.

### 2.4. Quality assessment

Each trial was independently assessed by two evaluators. The Cochrane Risk of Bias (RoB) tool was used to assess the bias risk of RCTs. Prospective cohort studies (PCSs) and retrospective cohort studies (RCSs) were evaluated by Newcastle–Ottawa scale (NOS) (27).

### 2.5. Statistical analysis

Cochrane Review Manager (version 5.3) was used to analyze the data for this research. Risk ratios (RR) and 95% confidence intervals (CI) are used to show the results of categorical variables. For continuous variables, the weighted mean difference (WMD) and 95% CI were used. When the study's continuous variables were means (range) or medians (IQR), the standard deviations were calculated using Luo's approach (28). Due to the differences in patient characteristics across studies, a random-effects model was preferred. The degree of statistical heterogeneity was estimated by Cochran's chi-square test and *I*^2^. We defined *I*^2^ values of 25%, 50%, and 75% as low, moderate, and high heterogeneity (29). Due to the small number of studies included, funnel plot and Begg's and Egger's test were not used to test the studies' deviation. The included studies were excluded one by one for sensitivity analysis.

## 3. Results

### 3.1. Study selection and quality assessment and risk of bias

**Figure 1:**
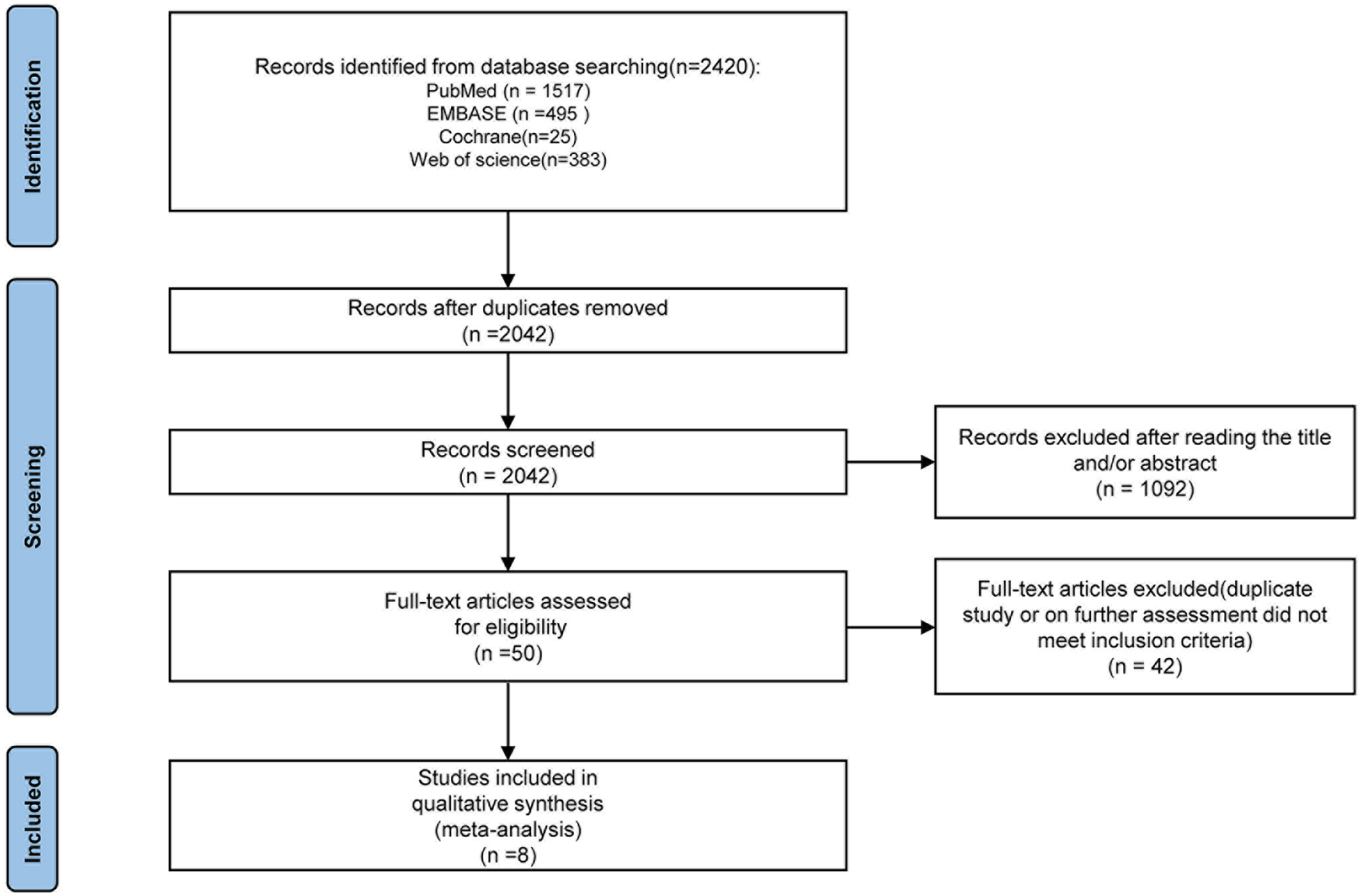
Flow chart illustrating summary of literature search results.

According to the pre-established search strategy, a total of eight studies were included in the final discussion (Figure 1), including two RCT and six cohort studies (PCS: 2, RCS: 4) (6, 20–26). The cohort studies were evaluated using modified NOS. Six studies scored 7–9 points and were indicated to be of high quality. Risk of bias assessment for RCTs is shown in Table 1.

**Table 1:** Risk of bias assessment of the randomized controlled trial.

Study	Random sequence generation	Allocation concealment	Blinding of participants and personnel	Blinding of outcome assessment	Incomplete outcome data	Selective reporting	Other bias
Li 2021	Unclear risk	Unclear risk	High risk	Low risk	Unclear risk	Low risk	High risk
Zhang 2021	Low risk	High risk	Unclear risk	Low risk	Unclear risk	Unclear risk	Unclear risk

### 3.2. Characteristics of included studies

Finally, we included eight studies with a total of 583 patients, of which 313 were in the ARSN-assisted PN group (AR group) and 270 were in the conventional PN group (NAR group). The baseline characteristics of the included studies are summarized in Table 2. The retroperitoneal approach was employed in all PN procedures. Sex, age, BMI, Padua score, R.E.N.A.L score and tumor size were comparable between the two groups of patients in all studies (Table 2).

**Table 2:** Characteristics of the studies included in the meta-analysis.

Study	Country	Number of patients	Sex F/M	*p* value	Age	*p* value	BMI	*p* value	Tumour size	*p* value	PADUA score	*p* value	R.E.N.A.L score	*p* value	Surgical technique	Study design	NOS
Zhang 2021	China	T:15 C:15	T:8/7 C:9/6	0.71	T:52.4 ± 11.8 C:50.5 ± 10.2	0.64	T:21.5 ± 1.5 C:22.5 ± 1.3	0.06	None	None	None	None	None	None	LPN	RCT	None
Wang 2015	China	T:21 C:14	T:11/10 C:8/6	0.89	T:58.5 (48, 73) C:62.1 (47, 80)	0.23	T:26.2 (22.5, 30.2) C:25.2 (23.0, 26.9)	0.39	T:29 (13, 44) C:34 (14, 42)	0.10	T:8.1 (6, 10) C:8.7 (7, 10)	0.11	T:7.0 (4, 9) C:6.9 (5, 8)	0.73	LPN	RCS	8
Satoshi 2020	Japan	T:42 C:42	T:31/11 C:30/12	0.87	T:58 (49–68) C:58 (42–67)	0.55	T:24.8 (22.5–26.8) C:24 (21.6–24)	0.60	T:27 (20–35) C:27.5 (20–36)	0.55	None	None	T:7 (5–8) C:7 (6–8)	1	RAPN	RCS	9
Porpiglia 2020	Italy	T:48 C:43	T:35/13 C:33/10	0.86	T:62 ± 15 C:58 ± 9.8	0.14	T:24.1 ± 3.7 C:25.9 ± 3.8	0.02	T:48.57 ± 18.67 C:44.6 ± 13.1	0.24	T:11 (10–12) C:10 (10–11)	0.65	None	None	RAPN	RCS	7
Porpiglia 2018	Italy	T:21 C:31	T:15/6 C:23/8	0.92	T:60.8 ± 12.3 C:59.5 ± 10.6	0.66	T:24 (23.5–25.5) C:25 (23.5–25)	0.24	T:50.8 ± 16.1 C:50.9 ± 15.1	0.97	T:11 (10–11) C:10.5 (10–11)	0.85	None	None	RAPN	PCS	7
Li 2022	China	T:16 C:25	T:10/6 C:14/11	0.68	T:45.8 ± 13.0 C:56.1 ± 12.4	0.39	T:23.8 ± 3.3 C:25.7 ± 4.3	0.40	T:46.3 ± 21.6 C:47.6 ± 36.2	0.07	T:9.5 (8.25–11) C:9.5 (8.25–11)	0.78	T:8 (6–9) C:7 (6–10)	0.22	RAPN	RCS	8
Li 2021	China	T:100 C:50	T:67/33 C:37/13	0.38	T:56.35 ± 13.47 C:55.6 ± 13.4	0.75	T:26.35 ± 4.15 C:27.2 ± 4.5	0.26	None	None	None	None	T:8.3 ± 1.25 C:8.6 ± 1.6	0.64	LPN	RCT	None
Li 2020	China	T:50 C:50	T:31/19 C:35/15	0.40	T:54.3 ± 12.1 C:56.9 ± 14.7	0.34	T:28.4 ± 1.4 C:28.5 ± 1.6	0.74	None	None	None	None	T:8.9 ± 1.9 C:8.4 ± 1.6	0.16	LPN	PCS	8

T, AR group; C, NAR group; PCS, prospective comparative studies; RCS, retrospective comparative studies; RCT, randomized controlled trial; BMI, body mass index; NOS, Newcastle–Ottawa Scale; None, not available.

### 3.3. Outcome

#### 3.3.1. EBL

**Figure 2:**
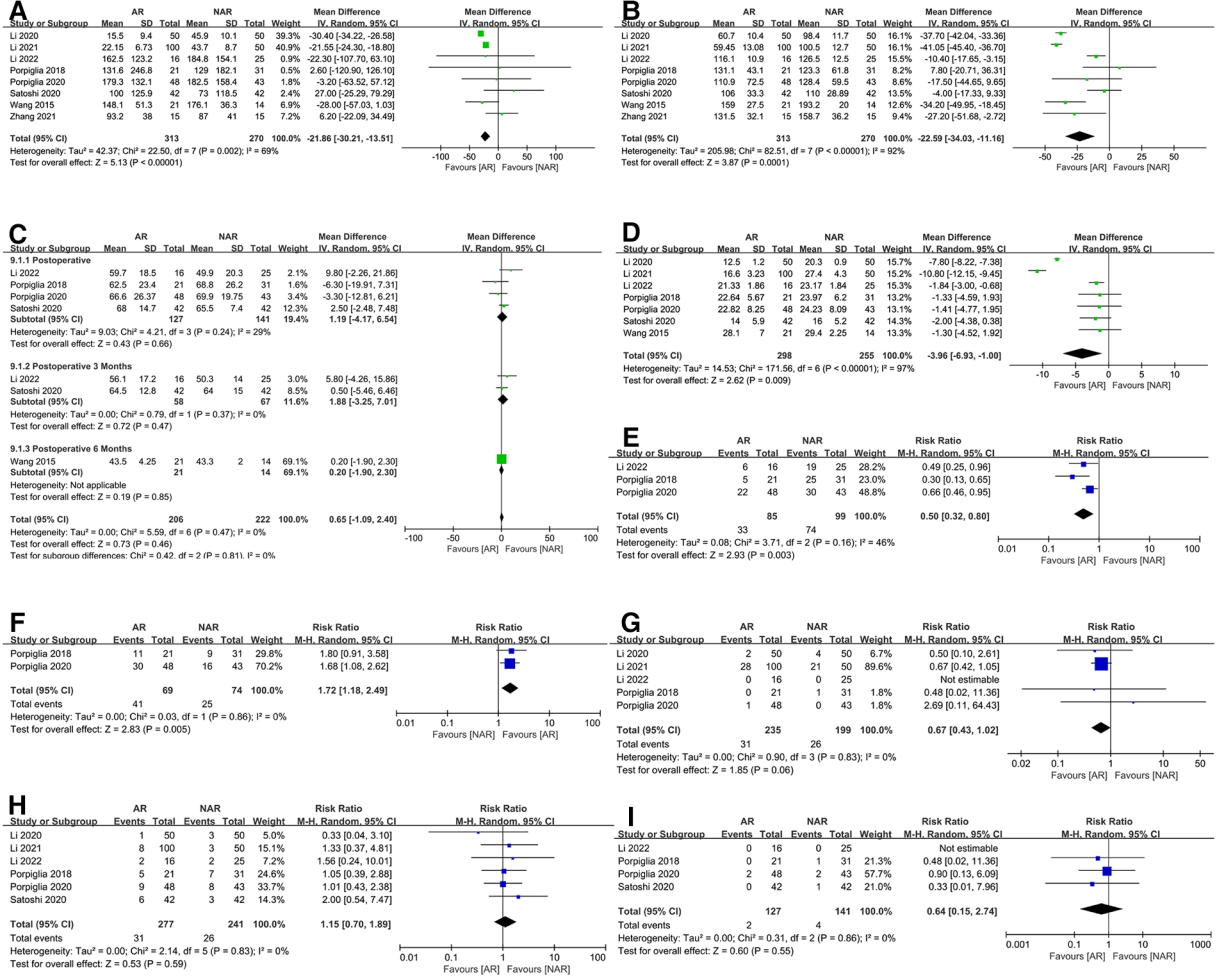
Forest plots for (**A**) estimated blood loss; (**B**) operative time; (**C**) perioperative eGFR; (**D**) warm ischemic time; (**E**) the rate of global ischemic; (**F**) the rate of enucleation; (**G**) the rate of conversion to RN; (**H**) postoperative complication; (**I**) positive margin rate.

All studies reported EBL (6, 20–26). We discovered that the EBL of the AR group was significantly lower than that of the NAR group. The high heterogeneity led to the adoption of a random effects model (MD = −21.86; 95% CI: −30.21, −13.51; *p* < 0.00001; *I*^2 ^= 69%, Figure 2A).

#### 3.3.2. OT

All studies provided data on OT (6, 20–26). The Meta-analysis suggested that the application of ARSN could significantly reduce the OT. However, there was high heterogeneity in the results (MD = −22.59; 95% CI: −34.03, −11.16; *p* = 0.0001; *I*^2 ^= 92%, Figure 2B).

#### 3.3.3. eGFR

Five studies published data on perioperative eGFR (6, 20, 21, 23, 26). Four of these studies reported postoperative eGFR (6, 21, 23, 26), two of which reported postoperative eGFR at 3 months (23, 26), and one of which reported postoperative eGFR at 6 months (20). The result showed no significant difference in eGFR between the AR and NAR groups at all postoperative periods (MD = 0.65; 95% CI: −1.09, 2.40; *p* = 0.46; *I*^2 ^= 0%, Figure 2C).

#### 3.3.4. WIT and global ischemia

We analyzed the WIT in six studies (6, 20–24, 26) and showed that the application of ARSN significantly shortened this factor. However, the heterogeneity test revealed high heterogeneity in the results (MD = −3.96; 95% CI: −6.93, −1.00; *p* = 0.009; *I*^2^ = 97%, Figure 2D). In terms of ischemia, three studies reported ischemic protocol (6, 21, 26), and we found a lower global ischemia rate in the AR group (RR = 0.50; 95% CI: 0.32, 0.80; *p* = 0.003; *I*^2 ^= 46%, Figure 2E).

#### 3.3.5. Enucleation and conversion to RN

Two studies by Porpiglia et al. (6, 21) described the rate of enucleation, and the result showed that the proportion of enucleation was higher in the AR group (RR = 1.72; 95% CI: 1.18, 2.49; *p* = 0.005; *I*^2^ = 0%, Figure 2F). The rate of conversion to RN was recorded in five studies (6, 21, 22, 24, 26). Overall, there was no substantial difference between the AR and NAR groups (RR = 0.67; 95% CI: 0.43, 1.02; *p* = 0.06; *I*^2^ = 0%, Figure 2G).

#### 3.3.6. Postoperative complication and positive margin rate

Six studies provided data on the rate of postoperative complications (6, 21–24, 26), and the results revealed no difference between the two groups (MD = 1.15; 95% CI: 0.70, 1.89; *p* = 0.59; *I*^2 ^= 0%, Figure 2H). Only four robot-assisted partial nephrectomy (RAPN) studies reported positive surgical margins (6, 21, 23, 26). The results showed no difference between the two groups (RR = 0.64; 95% CI: 0.15, 2.74; *p* = 0.55; *I*^2^ = 0%, Figure 2I).

#### 3.3.7. Subgroup analysis

We performed subgroup analyses due to the high heterogeneity. Subgroup analyses were based on the different surgical techniques and their associated risk: Laparoscopic partial nephrectomy (LPN)/RAPN subgroup and Low-Risk/High-Risk subgroup. Padua and R.E.N.A.L scores are valid indicators to assess the complexity of PN (30). According to the study by Li et al. and Porpiglia et al. (6, 24), we classified Padua ≥10 or R.E.N.A.L score >7 as high risk for surgery and below-threshold values as low risk. Except for the study by Zhang et al. (25), which did not provide score data, only the studies by Wang et al. and Satoshi et al. (20, 23) were deemed to be in the low-risk subgroup.

**Figure 3:**
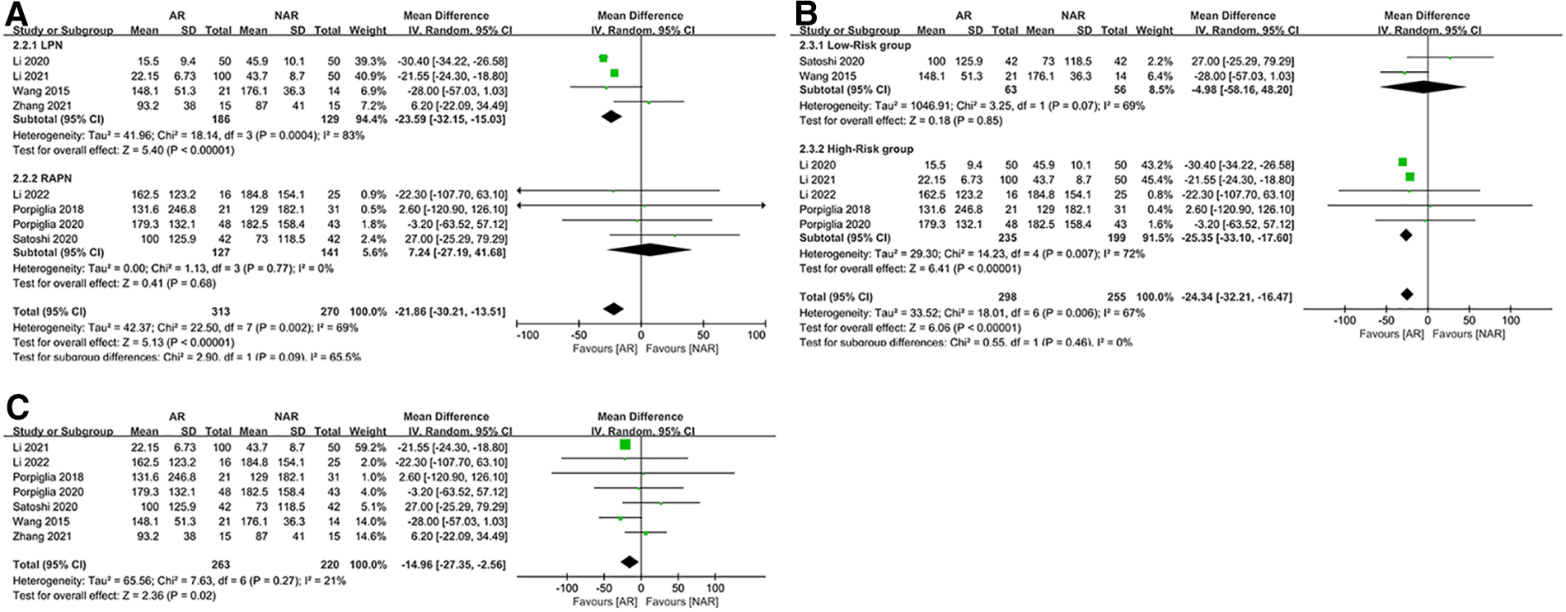
(**A**) Subgroup analysis by surgical technique for EBL; (**B**) subgroup analysis performed by the risk of the surgery for EBL; (**C**) EBL excluding Li 2020.

We performed a subgroup analysis of EBL. The results showed that heterogeneity was mainly in the LPN subgroup (MD = −23.59; 95% CI: −32.15, −15.03; *p* < 0.00001; *I*^2 ^= 83%, Figure 3A). There was no heterogeneity in the RAPN subgroup (MD = 7.24; 95% CI: −27.19, 41.68; *p* = 0.68; *I*^2 ^= 0%, Figure 3A). In addition, the EBL results in the LPN subgroup were consistent with the overall results, whereas, in the RAPN subgroup, there was no statistical difference in EBL between the AR and NAR groups (*p* = 0.68, Figure 3A). According to the result of the subgroup analysis of surgical risk, there was high heterogeneity in both subgroups and no statistical difference between subgroups (*p* = 0.46, Figure 3B). We observed that the result of EBL in the high-risk subgroup was consistent with the overall result, but in the low-risk subgroup, there was no statistical difference in EBL between the AR and NAR groups (MD = −4.98; 95% CI: −58.16, 48.20; *p* = 0.85; *I*^2 ^= 69%, Figure 3B). The study by Li et al. (22) contributed the most heterogeneity in this meta-analysis. After excluding this study, *I*^2^ dropped to 21%, while the result of EBL did not change (MD = −14.96; 95% CI: −27.35, −2.56; *p* = 0.02; *I*^2 ^= 21%, Figure 3C).

**Figure 4:**
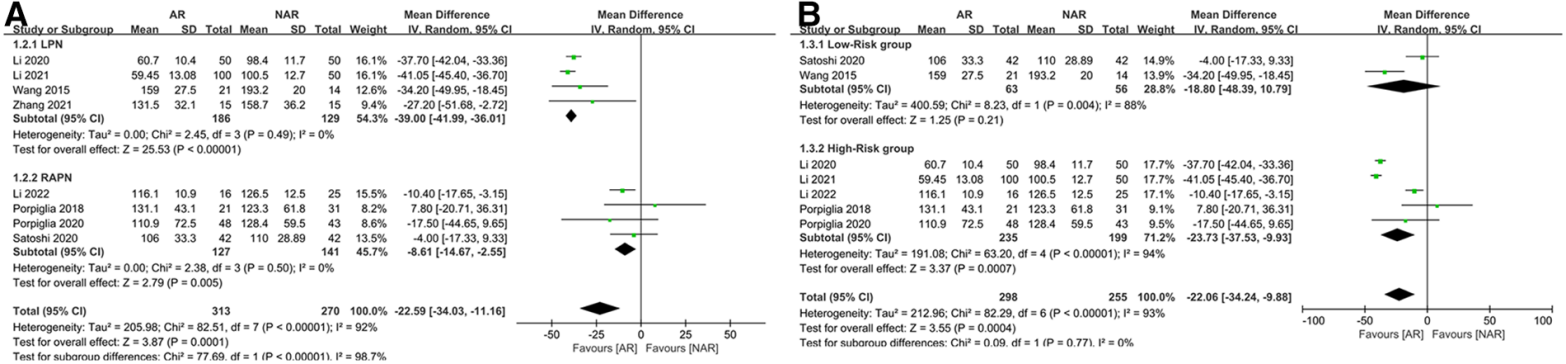
(**A**) Subgroup analysis by surgical technique for OT; (**B**) subgroup analysis performed by the risk of the surgery for OT.

For OT, subgroup analysis based on surgical technique showed that heterogeneity was mainly from differences in surgical techniques (*p* < 0.00001, Figure 4A), with no heterogeneity present within either LPN or RAPN subgroup. The result of OT in both subgroups was consistent with the overall result. The results of the subgroup analysis based on surgical risk showed that surgical risk was not a source of heterogeneity (*p* = 0.77, Figure 4B), with high heterogeneity within both subgroups. The result for OT in the high-risk subgroup was consistent with the overall result, but in the low-risk subgroup, there was no statistical difference in OT between the AR and NAR groups (MD = −18.80; 95% CI: −48.39, 10.79; *p* = 0.21; *I*^2 ^= 88%, Figure 4B).

**Figure 5:**
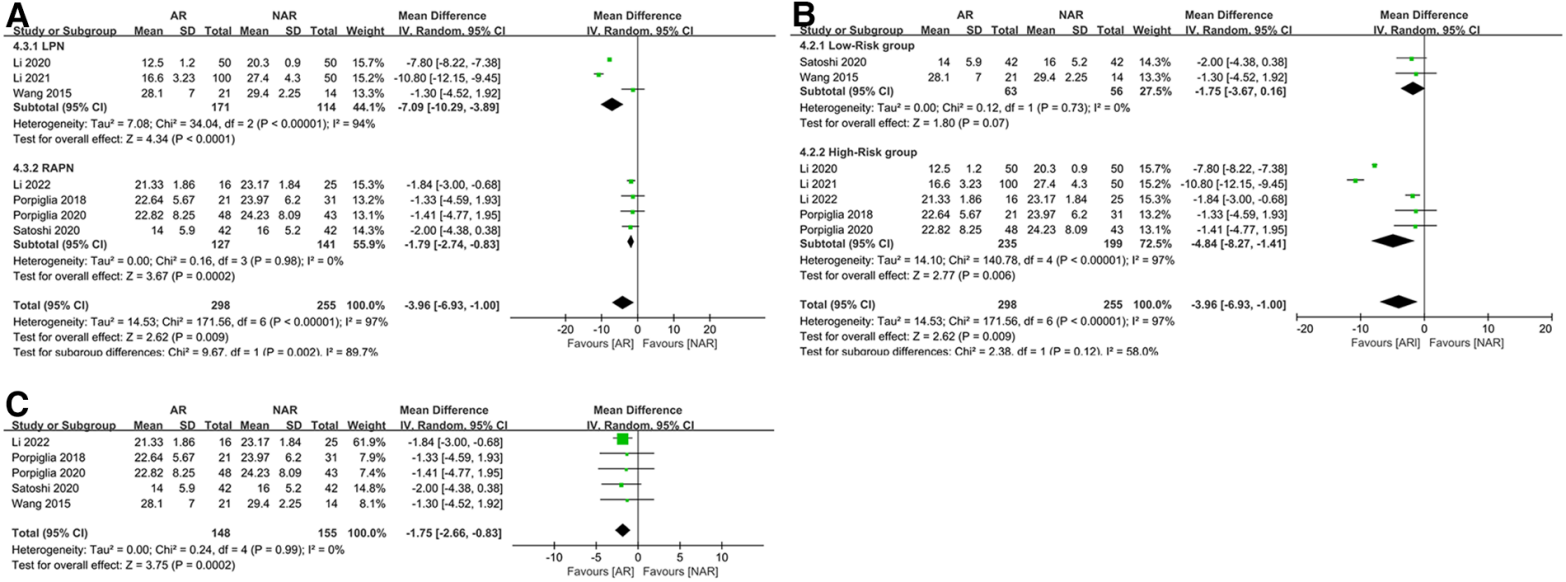
(**A**) Subgroup analysis by surgical technique for WIT; (**B**) subgroup analysis performed by the risk of the surgery for WIT; (**C**) WIT excluding Li 2020 and Li 2021.

For WIT, subgroup analysis based on surgical techniques showed that the LPN subgroup was the main source of heterogeneity (MD = −7.09; 95% CI: −10.29, −3.89; *p* < 0.0001; *I*^2^ = 94%, Figure 5A), and no heterogeneity existed in the RAPN subgroup (MD = −1.79; 95% CI: −2.74, −0.83; *p* = 0.0002; *I*^2^ = 0%, Figure 5A). The results of WIT in both subgroups were consistent with the overall result. Subgroup analysis based on surgical risk indicated high heterogeneity in the high-risk subgroup (MD = −4.84; 95% CI: −8.27, −1.41; *p* = 0.006; *I*^2^ = 97%, Figure 5B) and no heterogeneity in the low-risk subgroup (MD = −1.75; 95% CI: −3.67, −0.16; *p* = 0.07; *I*^2^ = 0%, Figure 5B). The result of WIT in the high-risk subgroup was consistent with the overall result, but in the low-risk subgroups, there was no statistical difference in WIT between the AR and NAR groups (*p* = 0.07, Figure 5B). After excluding two studies from the same author (Li 2020 and Li 2021) (22, 24), *I*^2^ decreased to 0 and the result of WIT remained unchanged (MD = −1.75; 95% CI: −2.66 −0.83; *p* = 0.0002; *I*^2^ = 0%, Figure 5C).

#### 3.3.8. Sensitivity analysis

We performed a sensitivity analysis of the results by excluding the individual experiments showing substantial heterogeneity. None of the study removals from the model showed an effect on our preceding main conclusions.

## 4. Discussion

According to GOBALCAN 2020, renal cancer accounted for 2.2% of all new cancer cases in 2020, as well as 1.8% of cancer deaths (31). NSS is the preferred treatment for stage cT1a renal tumors and one of the interventions for stage cT1b and cT2 renal tumors (32). Compared to RN, patients undergoing PN usually have a better prognosis for renal function and a lower risk of chronic kidney disease (CKD) (1). The key factors influencing the prognosis of patients undergoing PN are surgical margin, renal parenchyma volume preservation, and ischemia time. In PN, ensuring the negative surgical margin has the highest priority, which is directly related to the outcome of the treatment. For patients with a single kidney, multifocal masses, and other potential risks for future CKD, postoperative renal function should be improved by preserving as much renal parenchyma as possible and reducing renal damage due to ischemia (1).

Traditional DICOM format (CT/MRI) pictures are two-dimensional and shown in monotone grayscale, making it impossible to directly view complex anatomical features. For that, surgeons are required to mentally reconstruct these two-dimensional pictures into three-dimensional images. However, this largely depends on individual capabilities and expertise among surgeons, therefore the “imaging reconstruction” can be unstable and consequently unreliable. In contrast, utilizing AR technology, the surgeon could directly observe the critical structures in the surgery area by zooming in, rotating, and altering the transparency with a 3D model of the kidney (12, 13). Our findings suggest that utilizing ARSN in PN could significantly reduce OT, which might benefit from detailed preoperative planning and intraoperative visualization of renal anatomy based on 3D models. Moreover, 3D model-based preoperative virtual surgery allow surgeons to familiarize themselves with surgical procedures in advance. We discovered that using ARSN significantly reduced EBL. In ARSN-based PN, surgeons had immediate access to anatomical information about the tumor location and surrounding tissues, which allowed them to perform purposeful tumor excision while avoiding unneeded intrusive procedures. Through subgroup analysis, we observed that this difference was not significant in RAPN. This could be due to the surgical equipment's benefits obscuring the effect of ARSN.

The amount of parenchyma saved is the most critical factor influencing the recovery of renal function after PN (33). Greater resection volume is frequently linked to a worse prognosis for renal function following surgery in patients with complicated renal malignancies (1). Enucleation is a surgical treatment strategy that can maximize the preservation of normal renal tissue. Enucleation is effective in treating small renal lesions, although it can be challenging to determine safe boundaries (34–36). Here, we noticed that enucleation was more commonly employed in the AR group, which may benefit from the application of high-precision three-dimensional images of the kidney in the surgical procedures (6, 21). The utilization of ARSN could broaden the indications for PN and improve the success rate of NSS surgery compared to the conventional surgical approach (24). However, there was no significant difference in the probability of conversion to RN between the two groups in this study. That might owe to the fact that patients were screened for inclusion in the trials.

In addition to parenchymal mass preservation, Ischemia duration is also an important factor affecting postoperative renal function recovery after PN (1, 33). Although the threshold of warm ischemia duration that causes substantial impairment to renal function is unclear, it is widely accepted that WIT of less than 25–30 min has little effect on renal function (37, 38). However, it is still suggested that the early release of warm ischemia is more beneficial for functional recovery (39–41). We found that AR navigation significantly reduced WIT, which may be due to the visual display of the renal mass and vessels, reducing intraoperative exploration time and making it easier to determine the boundaries of resection. Compared with reducing the time of ischemia, avoiding ischemia is a more direct means. Traditional PN surgery frequently employs Hilar occlusion to obtain a safe and effective view, although this results in global ischemia. However, studies in recent years have indicated that Hilar occlusion is not required in PN (42–44). Studies by Smith et al. and Desai et al. (42, 43) have shown that selective clamping could better preserve renal function without compromising the oncological efficacy. However, selective clamping and zero ischemia require a more detailed dissection of the branch vessels, which makes these techniques more challenging. Three-dimensional CT reconstruction of renal vessels, intraoperative color Doppler ultrasonography, and near-infrared fluorescence imaging were the main employed techniques in clinical practice to guide selective clamping. Here, we analyze the application of selective clamping and zero ischemia in PN based on ARSN and conventional navigation (IOUS, CT images, etc.). The result showed that a lower rate of patients underwent global ischemia in the AR group compared with the NAR group. Therefore, ARSN may provide more aid for PN surgery than conventional methods.

In terms of renal function, we found no statistically significant difference between the two groups. However, the amount of preserved renal parenchyma was theoretically closely associated with the eventual level of postoperative eGFR (45). The compensatory effect of contralateral healthy and remnant kidneys and the lack of long-term eGFR monitoring may lead to masked differences in renal function between the two groups. In isolated renal patients, Li et al. (26) discovered that patients in the AR group had lower levels of reduced eGFR than those in the NAR group. Furthermore, Porpiglia et al. (6) used renal scintigraphy to assess the effective renal plasmatic flow of operated kidneys and found that patients in the AR group performed better. As a result, we conclude that ARSN has a beneficial effect on renal function recovery in patients receiving PN.

There was no statistically significant difference in the rates of positive surgical margins and postoperative complications between the AR and NAR groups, which may be due to the surgeon's caution and extensive experience in NSS. Larcher et al. (46) and Porpiglia et al. (47) evaluated the learning curve of NSS and discovered that the rate of complications steadily declined and renal ischemia improved as the number of surgical cases increased. When the image-guided system was not utilized, it took a long time to reach the learning curve (46). In contrast, ARSN could provide surgeons with reliable intraoperative guidance while shortening the formidable learning curve required for NSS (48).

The reconstruction and intraoperative registration of AR models in all studies were performed manually. Studies have shown that the constructed 3D models of the kidney can objectively and realistically reflect the anatomical features of the kidney (6, 20–26). Intraoperative manual registration of the models can also achieve the required clinical accuracy (6, 20–26). The augmented reality techniques used across studies were not fundamentally different. Differences in processes may lead to high heterogeneity in results, but after sensitivity analysis, we found that such differences do not affect the final results. Concerning the cost of ARSN, one study reported that a higher amount of money (additional 500–600 dollars for the engineers) together with labor costs were unavoidable due to the manual involvement required for 3D model reconstruction and intraoperative model registration (26). The application of automated registration may reduce these additional costs in the future, thanks to advances in artificial intelligence technology (49).

This study has several limitations. First, the included studies were few and primarily retrospective, with only two RCTs reporting limited data, which may have resulted in confounding factors that could not be eradicated. Second, because our data were mainly from patients with complex renal neoplasms, our conclusions may be more applicable to these patients. Third, the application of ARSN is still in its early stages and lacks a uniform and standardized approach; therefore, the procedures of ARSN used in all of the selected research differ, which may lead to the high heterogeneity of some results.

## 5. Conclusion

In summary, the application of ARNS in NSS can effectively reduce intraoperative blood loss, shorten the duration of operation, and reduce damage to normal renal tissue. Although there is no direct evidence, our results still show that AR navigation technology has potential advantages in improving the recovery of renal function after PN. It is worth noting that the application of augmented reality technology in surgery is still in the early stage of exploration, and the research we report has the limitations of a rigid 3D model and manual registration technology. In the future, more intelligent and accurate AR navigation technologies may be able to achieve better clinical results in PN. Until then, we need high-quality RCTs to further improve the credibility of the conclusions.

## Data Availability

The original contributions presented in the study are included in the article/Supplementary Material, further inquiries can be directed to the corresponding author.
